# *QuickStats:* Birth Rates* for Females Aged 15–19 Years, by Age Group — National Vital Statistics System, United States, 1991–2021

**DOI:** 10.15585/mmwr.mm7203a8

**Published:** 2023-01-20

**Figure:**
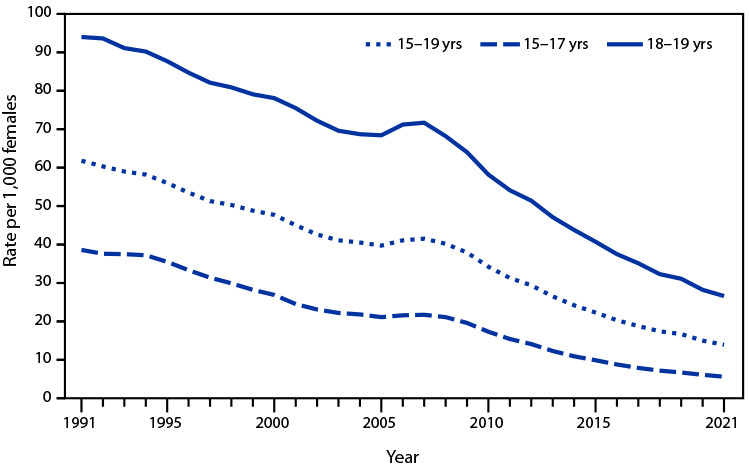
The birth rate for females aged 15–19 years declined from a 1991 peak of 61.8 per 1,000 females to a record low of 13.9 in 2021. From 1991 to 2021, the rate for females aged 15–17 years declined from 38.6 to 5.6 and from 94.0 to 26.6 for those aged 18–19 years. Most of the decline occurred during 2007–2021, with rates down 67% for females aged 15–19 years, 74% for females aged 15–17 years, and 63% for females aged 18–19 years. During 1991–2021, decreases in birth rates for females aged 15–17 years were larger than the decreases for those aged 18–19 years.

**Source:** National Center for Health Statistics, National Vital Statistics System, Natality Data, 2021. https://www.cdc.gov/nchs/nvss/births.htm

